# Supplementary material for: 1H LF-NMR Self-Diffusion Measurements for Rapid Monitoring of an Edible Oil’s Food Quality with Respect to Its Oxidation Status
Source: Molecules. 2022 Sep 16;27(18):6064. doi: 10.3390/molecules27186064 (PMC9505792; doi:10.3390/molecules27186064)
Supplement: Supplementary file 1 [file molecules-27-06064-s001.zip › molecules-1900325-supplementary.pdf]

molecules

MDPI

Supplemental Material:

Table S1: Summary of diffusion coefficient, peroxide value and *Para*-Anisidine value of Linseed oil (LSO) at different times of oxidation.

Oxidation starting date	Ox time	sample	Diffusion Coefficient (D)	Peroxide Value (PV)	*Para*-Anisidine Value (PAV)	TOTOX
14.11.21	0	1	0.037	3.02	1.54	7.58
14.11.21	0	2	0.038	2.62	1.54	6.78
14.11.21	0	3	0.044	2.77	1.54	7.08
14.11.21	0	4	0.045	2.24	1.54	6.02
14.11.21	0	5	0.045	2.88	1.54	7.3
14.11.21	0	6	0.047	3.28	1.54	8.1
14.11.21	0	7	0.038	2.68	1.54	6.9
14.11.21	0	8	0.040	3.18	1.54	7.9
14.11.21	0	9	0.038	2.65	1.54	6.84
14.11.21	0	10	0.037	2.68	1.54	6.9
14.11.21	24	1	0.038	19.39	13.99	52.77
14.11.21	24	2	0.040	27.9	13.99	69.79
14.11.21	24	3	0.038	25.32	13.99	64.63
14.11.21	48	1	0.040	78.61	92.85	250.07
14.11.21	48	2	0.033	72.59	92.85	238.03
14.11.21	48	3	0.033	59.76	92.85	212.37
14.11.21	72	1	0.028	69.22	110.14	248.58
14.11.21	72	2	0.028	84.98	110.14	280.1
14.11.21	72	3	0.024	60.62	110.14	231.38
14.11.21	96	1	0.030	147.27	120.84	415.38
14.11.21	96	2	0.026	143.65	120.84	408.14
14.11.21	96	3	0.024	126.92	120.84	374.68
14.11.21	120	1	0.018	184.42	125.05	493.89
14.11.21	120	2	0.020	141.63	125.05	408.31
14.11.21	120	3	0.017	119.07	125.05	363.19
21.11.21	0	1	0.042	3.02	0.64	6.68
21.11.21	0	2	0.038	2.62	0.64	5.88
21.11.21	0	3	0.042	2.77	0.64	6.18
21.11.21	0	4	0.041	2.24	0.64	5.12
21.11.21	0	5	0.042	2.88	0.64	6.4
21.11.21	0	6	0.044	3.28	0.64	7.2
21.11.21	0	7	0.039	2.68	0.64	6
21.11.21	0	8	0.040	3.18	0.64	7
21.11.21	0	9	0.047	2.65	0.64	5.94
21.11.21	0	10	0.044	2.68	0.64	6
21.11.21	24	1	0.041	20.54	10.66	51.74
21.11.21	24	2	0.039	13.39	10.66	37.44
21.11.21	24	3	0.037	19.87	10.66	50.4
21.11.21	24	4	0.040	16.87	10.66	44.4
21.11.21	24	5	0.037	16.35	10.66	43.36
21.11.21	24	6	0.044	22.7	10.66	56.06
21.11.21	24	7	0.041	17.22	10.66	45.1
21.11.21	24	8	0.035	18.94	10.66	48.54
21.11.21	24	9	0.038	18.48	10.66	47.62
21.11.21	24	10	0.045	16.13	10.66	42.92

21.11.21	48	1	0.035	58.77	50.56	168.1
21.11.21	48	2	0.041	56.44	50.56	163.44
21.11.21	48	3	0.040	50	50.56	150.56
21.11.21	48	4	0.036	43.74	50.56	138.04
21.11.21	48	5	0.032	36.32	50.56	123.2
21.11.21	48	6	0.029	40.23	50.56	131.02
21.11.21	48	7	0.032	56.38	50.56	163.32
21.11.21	48	8	0.045	54.77	50.56	160.1
21.11.21	48	9	0.035	42.48	50.56	135.52
21.11.21	48	10	0.029	46.8	50.56	144.16
21.11.21	72	1	0.034	75	85.72	235.72
21.11.21	72	2	0.038	41.73	85.72	169.18
21.11.21	72	3	0.032	51.94	85.72	189.6
21.11.21	72	4	0.037	70	85.72	225.72
21.11.21	72	5	0.035	75.84	85.72	237.4
21.11.21	72	6	0.033	78.57	85.72	242.86
21.11.21	72	7	0.033	85.98	85.72	257.68
21.11.21	72	8	0.038	94.03	85.72	273.78
21.11.21	72	9	0.034	94.88	85.72	275.48
21.11.21	72	10	0.036	93.62	85.72	272.96
21.11.21	96	1	0.030	109.11	86.83	305.05
21.11.21	96	2	0.031	119.17	86.83	325.17
21.11.21	96	3	0.033	129.16	86.83	345.15
21.11.21	96	4	0.033	112.84	86.83	312.51
21.11.21	96	5	0.031	93.16	86.83	273.15
21.11.21	96	6	0.032	118.91	86.83	324.65
21.11.21	96	7	0.041	96.77	86.83	280.37
21.11.21	96	8	0.032	134	86.83	354.83
21.11.21	96	9	0.028	117.8	86.83	322.43
21.11.21	96	10	0.030	132.64	86.83	352.11
21.11.21	120	1	0.029	104.71	88.04	297.46
21.11.21	120	2	0.026	121.72	88.04	331.48
21.11.21	120	3	0.029	103.83	88.04	295.7
21.11.21	120	4	0.025	125.18	88.04	338.4
21.11.21	120	5	0.030	141.11	88.04	370.26
21.11.21	120	6	0.028	129.02	88.04	346.08
21.11.21	120	7	0.026	121.64	88.04	331.32
21.11.21	120	8	0.023	139.5	88.04	367.04
21.11.21	120	9	0.032	133.39	88.04	354.82
21.11.21	120	10	0.029	138.68	88.04	365.4
28.11.21	0	1	0.037	1.99	1.67	5.65
28.11.21	0	2	0.038	1.68	1.67	5.03
28.11.21	0	3	0.044	2.22	1.67	6.11
28.11.21	24	1	0.039	92.77	34.41	219.95
28.11.21	24	2	0.042	86.55	34.41	207.51
28.11.21	24	3	0.039	78.02	34.41	190.45
28.11.21	48	1	0.036	156.89	73.78	387.56
28.11.21	48	2	0.032	168.32	73.78	410.42
28.11.21	48	3	0.031	166.2	73.78	406.18
28.11.21	72	1	0.025	179.87	93.16	452.9
28.11.21	72	2	0.024	200.9	93.16	494.96
28.11.21	72	3	0.026	192.84	93.16	478.84
28.11.21	96	1	0.017	231.45	113.78	576.68
28.11.21	96	2	0.015	223.56	113.78	560.9

28.11.21	96	3	0.012	229.25	113.78	572.28
5.12.21	0	1	0.040	2.2	2.080	6.48
5.12.21	0	2	0.039	2.54	2.080	7.16
5.12.21	0	3	0.036	2.41	2.080	6.9
5.12.21	24	1	0.036	12.3	5.410	30.01
5.12.21	24	2	0.045	13.1	5.410	31.61
5.12.21	24	3	0.039	11.47	5.410	28.35
5.12.21	48	1	0.035	128.85	62.180	319.88
5.12.21	48	2	0.039	137.21	62.180	336.6
5.12.21	48	3	0.038	134.65	62.180	331.48
5.12.21	72	1	0.039	159.77	78.120	397.66
5.12.21	72	2	0.029	165.9	78.120	409.92
5.12.21	72	3	0.030	143.66	78.120	365.44
5.12.21	96	1	0.024	188.97	126.150	504.09
5.12.21	96	2	0.030	258.88	126.150	643.91
5.12.21	96	3	0.022	196.5	126.150	519.15
5.12.21	120	1	0.015	218.690	132.740	570.12
5.12.21	120	2	0.016	216.140	132.740	565.02
5.12.21	120	3	0.010	182.740	132.740	498.22
12.12.21	0	1	0.039	1.95	1.71	5.61
12.12.21	0	2	0.042	2.43	1.71	6.57
12.12.21	0	3	0.039	2.45	1.71	6.61
12.12.21	24	1	0.037	60.13	24.2	144.46
12.12.21	24	2	0.041	63.97	24.2	152.14
12.12.21	24	3	0.040	61.18	24.2	146.56
12.12.21	48	1	0.035	162.84	123.53	449.21
12.12.21	48	2	0.032	162	123.53	447.53
12.12.21	48	3	0.033	136.69	123.53	396.91
12.12.21	72	1	0.026	174.81	135.26	484.88
12.12.21	72	2	0.028	171.04	135.26	477.34
12.12.21	72	3	0.028	169.87	135.26	475
12.12.21	96	1	0.017	195.22	139.01	529.45
12.12.21	96	2	0.023	178.38	139.01	495.77
12.12.21	96	3	0.015	149.9	139.01	438.81
12.12.21	120	1	0.012	137.61	150.07	425.29
12.12.21	120	2	0.013	161.86	150.07	473.79
12.12.21	120	3	0.012	169.11	150.07	488.29
19.12.21	0	1	0.046	2.17	1.59	5.93
19.12.21	0	2	0.047	1.51	1.59	4.61
19.12.21	0	3	0.045	1.99	1.59	5.57
19.12.21	24	1	0.044	14.13	6.58	34.84
19.12.21	24	2	0.045	15.6	6.58	37.78
19.12.21	24	3	0.046	19.12	6.58	44.82
19.12.21	48	1	0.031	112.87	102.44	328.18
19.12.21	48	2	0.037	127.24	102.44	356.92
19.12.21	48	3	0.032	103.92	102.44	310.28
19.12.21	72	1	0.025	158.86	119.75	437.47
19.12.21	72	2	0.025	141.65	119.75	403.05
19.12.21	72	3	0.030	169.52	119.75	458.79
19.12.21	96	1	0.020	214.1	125.63	553.83
19.12.21	96	2	0.021	198.99	125.63	523.61
19.12.21	96	3	0.022	209.08	125.63	543.79
19.12.21	120	1	0.012	210.49	159.5	580.48
19.12.21	120	2	0.013	223.37	159.5	606.24

19.12.21	120	3	0.016	196.68	159.5	552.86
09.01.22	0	1	0.039	3.64	1.58	8.86
09.01.22	0	2	0.036	3.55	1.58	8.68
09.01.22	0	3	0.041	2.72	1.58	7.02
09.01.22	24	1	0.047	27.55	6.83	61.93
09.01.22	24	2	0.050	36.03	6.83	78.89
09.01.22	24	3	0.046	37.76	6.83	82.35
09.01.22	48	1	0.027	199.06	115.68	513.8
09.01.22	48	2	0.036	204.06	115.68	523.8
09.01.22	48	3	0.028	175.22	115.68	466.12
09.01.22	72	1	0.021	172.92	139.8	485.64
09.01.22	72	2	0.018	156.97	139.8	453.74
09.01.22	72	3	0.021	232.68	139.8	605.16
09.01.22	96	1	0.018	213.82	186.29	613.93
09.01.22	96	2	0.015	200.1	186.29	586.49
09.01.22	96	3	0.016	175.28	186.29	536.85
09.01.22	120	1	0.012	127.4	102.77	357.57
09.01.22	120	2	0.015	142.06	102.77	386.89
09.01.22	120	3	0.013	119.87	102.77	342.51
06.02.22	0	1	0.035	4.2	1.37	9.77
06.02.22	0	2	0.039	5.86	1.37	13.09
06.02.22	0	3	0.035	4.8	1.37	10.97
06.02.22	24	1	0.043	30.69	11.97	73.35
06.02.22	24	2	0.044	25.74	11.97	63.45
06.02.22	24	3	0.045	24.5	11.97	60.97
06.02.22	48	1	0.036	93.14	115.51	301.79
06.02.22	48	2	0.034	130.23	115.51	375.97
06.02.22	48	3	0.036	124.11	115.51	363.73
06.02.22	72	1	0.030	180.36	139.15	499.87
06.02.22	72	2	0.035	151.9	139.15	442.95
06.02.22	72	3	0.033	175.22	139.15	489.59
06.02.22	96	1	0.023	129.07	157.28	415.42
06.02.22	96	2	0.025	116.33	157.28	389.94
06.02.22	96	3	0.022	104.89	157.28	367.06
06.02.22	120	1	0.018	179.03	215.61	573.67
06.02.22	120	2	0.019	190.1	215.61	595.81
06.02.22	120	3	0.018	192.48	215.61	600.57

Table S2: Averages of D, PV, AV and TOTOX of all Linseed oil (LSO) samples

LSO - Average of all samples	Diffusion coefficient (D)	Peroxide Value (PV)	*Para*-Anisidine value (PAV)	TOTOX
0	0.0401	2.80	1.36	6.96
24	0.0413	31.09	13.44	75.62
48	0.0344	107.11	82.69	296.91
72	0.0300	134.25	106.90	375.41
96	0.0240	158.73	121.75	439.21
120	0.0195	156.13	126.34	438.60

Table S3: Summary of diffusion coefficient (D), peroxide value (PV) and *para*-anisidine value (PAV) of all other edible oils (soybean, canola, olive, coconut and butter) at different times of oxidation

Date	Oxidation time,h	sample	D	PV	PAV	TOTOX
16.01.2022	0	1	0.032±0.003	8.43	0.87	17.73
Canola oil	0	2	0.032±0.004	6.7	0.87	14.27
	0	3	0.025±0.005	6.79	0.87	14.45
	24	1	0.028±0.006	17.2	5.42	39.82
	24	2	0.027±0.002	16.77	5.42	38.96
	24	3	0.027±0.004	15.36	5.42	36.14
	48	1	0.029±0.003	54.05	26.48	134.58
	48	2	0.025±0.004	54.93	26.48	136.34
	48	3	0.030±0.002	60.35	26.48	147.18
	72	1	0.025±0.004	120.35	41.18	281.88
	72	2	0.022±0.002	96.86	41.18	234.9
	72	3	0.023±0.004	104.93	41.18	251.04
	96	1	0.020±0.005	202.98	157.45	563.41
	96	2	0.020±0.004	203.73	157.45	564.91
	96	3	0.017±0.004	212.87	157.45	583.19
	120	1	0.015±0.005	256.79	181.75	695.33
	120	2	0.020±0.005	245.78	181.75	673.31
	120	3	0.023±0.004	217.34	181.75	616.43
16.01.2022	0	1	0.029±0.005	7.61	8.9	24.12
Olive oil	0	2	0.029±0.002	5.87	8.9	20.64
	0	3	0.031±0.002	5.96	8.9	20.82
	24	1	0.031±0.004	17.2	10.27	44.67
	24	2	0.028±0.004	16.77	10.27	43.81
	24	3	0.025±0.004	15.36	10.27	40.99
	48	1	0.029±0.004	54.05	12.46	120.56
	48	2	0.023±0.004	55.76	12.46	123.98
	48	3	0.022±0.005	58.44	12.46	129.34
	72	1	0.025±0.004	120.35	12.9	253.6
	72	2	0.027±0.004	96.86	12.9	206.62
	72	3	0.021±0.005	104.93	12.9	222.76
	96	1	0.026±0.006	68.54	12.77	149.85
	96	2	0.023±0.003	57.63	12.77	128.03
	96	3	0.023±0.003	60.2	12.77	133.17
	120	1	0.026±0.004	130.03	34.09	294.15
	120	2	0.024±0.003	171.98	34.09	378.05
	120	3	0.027±0.004	163.72	34.09	361.53
23.01.2022	0	1	0.035±0.008	9.74	0.9	20.38
Coconut oil	0	2	0.038±0.007	7.11	0.9	15.12
	0	3	0.038±0.007	7.68	0.9	16.26
	24	1	0.034±0.005	6.46	1.2	14.12
	24	2	0.037±0.004	7.18	1.2	15.56
	24	3	0.042±0.006	7.45	1.2	16.1
	48	1	0.036±0.003	7.95	1.29	17.19
	48	2	0.037±0.004	8.22	1.29	17.73
	48	3	0.036±0.007	7.33	1.29	15.95
	72	1	0.038±0.008	6.27	1.38	13.92
	72	2	0.036±0.004	6.68	1.38	14.74
	72	3	0.034±0.005	6.66	1.38	14.7

	96	1	0.037±0.005	5.19	2.05	12.43
	96	2	0.030±0.005	5.78	2.05	13.61
	96	3	0.039±0.002	5.4	2.05	12.85
	120	1	0.038±0.003	5.8	2.63	14.23
	120	2	0.033±0.004	5.9	2.63	14.43
	120	3	0.040±0.005	6.62	2.63	15.87
23.01.2022	0	1	0.178±0.005	7.03	0.34	14.4
Margarine	0	2	0.197±0.006	6.1	0.34	12.54
	0	3	0.211±0.006	5.38	0.34	11.1
	24	1	0.031±0.004	10.03	0.8	20.86
	24	2	0.027±0.005	10.65	0.8	22.1
	24	3	0.024±0.005	8.45	0.8	17.7
	48	1	0.028±0.003	14.72	3	32.44
	48	2	0.028±0.004	17.16	3	37.32
	48	3	0.028±0.005	15.02	3	33.04
	72	1	0.027±0.004	19.63	4.17	43.43
	72	2	0.021±0.005	17.64	4.17	39.45
	72	3	0.024±0.006	20.58	4.17	45.33
	96	1	0.028±0.004	48.25	6.45	102.95
	96	2	0.032±0.006	68.38	6.45	143.21
	96	3	0.028±0.003	62.85	6.45	132.15
	120	1	0.021±0.004	274.27	78.32	626.86
	120	2	0.026±0.004	221.41	78.32	521.14
	120	3	0.022±0.003	423.05	78.32	924.42
30.01.2022	0	1	0.036±0.004	6.96	0.65	14.57
Soybeen oil	0	2	0.038±0.005	6.97	0.65	14.59
	0	3	0.034±0.004	8.21	0.65	17.07
	24	1	0.029±0.004	41.06	9.82	91.94
	24	2	0.027±0.006	39.84	9.82	89.5
	24	3	0.034±0.008	40.89	9.82	91.6
	48	1	0.032±0.003	109.04	31.94	250.02
	48	2	0.030±0.004	139.97	31.94	311.88
	48	3	0.031±0.003	122.15	31.94	276.24
	72	1	0.026±0.005	255.96	126.58	638.5
	72	2	0.024±0.005	202.23	126.58	531.04
	72	3	0.026±0.004	252.96	126.58	632.5
	96	1	0.016±0.003	232.28	233.56	698.12
	96	2	0.022±0.009	238.84	233.56	711.24
	96	3	0.021±0.005	241.75	233.56	717.06
	120	1	0.013±0.005	246.23	295.16	787.62
	120	2	0.015±0.005	289.03	295.16	873.22
	120	3	0.013±0.006	223.31	295.16	741.78
30.01.2022	0	1	0.147±0.005	6.33	1.12	13.78
Butter	0	2	0.166±0.005	6.6	1.12	14.32
	0	3	0.170±0.005	7.78	1.12	16.68
	24	1	0.028±0.006	9.32	1.39	20.03
	24	2	0.026±0.007	8.98	1.39	19.35
	24	3	0.024±0.004	9.13	1.39	19.65
	48	1	0.033±0.002	13.37	2.19	28.93
	48	2	0.035±0.003	11.97	2.19	26.13
	48	3	0.032±0.003	9.26	2.19	20.71
	72	1	0.031±0.004	8.97	2.33	20.27
	72	2	0.030±0.002	9.17	2.33	20.67

	72	3	0.026±0.003	7.46	2.33	17.25
	96	1	0.034±0.005	6.7	3.12	16.52
	96	2	0.030±0.004	7.06	3.12	17.24
	96	3	0.035±0.004	8.19	3.12	19.5
	120	1	0.029±0.006	8.24	4.84	21.32
	120	2	0.030±0.005	7.82	4.84	20.48
	120	3	0.028±0.005	9.93	4.84	24.7

Table S4: More detailed version if Table 1

**Time of heating (hr)**	**0**			**24**			**48**			**72**			**96**			**120**		
	PV (mmol/kg}	PAV (mmol/kg}	TOTOX (mmol/kg}	PV (mmol/kg}	PAV (mmol/kg}	TOTOX (mmol/kg}	PV (mmol/kg}	PAV (mmol/kg}	TOTOX (mmol/kg}	PV (mmol/kg}	PAV (mmol/kg}	TOTOX (mmol/kg}	PV (mmol/kg}	PAV (mmol/kg}	TOTOX (mmol/kg}	PV (mmol/kg}	PAV (mmol/kg}	TOTOX (mmol/kg}
**Saturated FA**																		
Butter	6.9 ±0.63	1.12	14.93 ±1.26	9.14 ±0.14	1.39	19.68 ±0.28	11.53 ±1.71	2.19	25.26 ±3.41	8.53 ± 0.76	2.33	19.4 ±1.53	7.32 ±0.63	3.12	17.75 ±1.27	8.66 ±0.91	4.84	22.17 ±1.82
Coconut oil	8.18 ±1.13	0.9	17.25 ±2.26	7.03 ±0.42	1.2	15.26 ±0.84	7.83 ±0.37	1.29	16.96 ±0.75	6.54 ± 0.19	1.38	14.45 ±0.38	5.46 ±0.24	2.05	12.96 ±0.49	6.11 ±0.37	2.63	14.84 ±0.73
**Mono-unsaturated FA**																		
Olive oil	6.48 ±0.8	8.9	21.86 ±1.6	16.44 ±0.79	10.27	43.16 ±1.57	56.08 ±1.81	12.46	124.63 ±3.61	107.38 ± 9.74	12.9	227.66 ±19.49	62.12 ±4.66	12.77	137.02 ±9.31	155.24 ±18.14	34.09	344.58 ±36.29
Canola oil	7.31 ±0.8	0.87	15.48 ±1.59	16.44 ±0.79	5.42	38.31 ±1.57	56.44 ±2.79	26.48	139.37 ±5.57	107.38 ± 9.74	41.18	255.94 ±19.49	206.53 ±4.5	157.45	570.5 ±8.99	239.97 ±16.62	181.75	661.69 ±33.24
**Poly-unsaturated FA**																		
Soy oil	7.38 ±0.59	0.65	15.41 ±1.17	40.6 ±0.54	9.82	91.01 ±1.08	123.72 ±12.68	31.94	279.38 ±25.35	237.05 ±24.65	126.58	600.68 ±49.3	237.62 ±3.96	233.56	708.81 ±7.92	252.86 ±27.24	295.16	800.87 ±54.47
Linseed oil	3.3 ± 0.41	1.58	8.19 ± 0.83	33.78 ± 4.46	6.83	74.39 ± 8.92	192.78 ± 12.58	115.68	501.24 ± 25.17	187.52 ± 32.59	139.8	514.85 ± 65.18	196.4 ± 15.95	186.29	579.09 ± 31.9	129.78 ± 9.21	102.77	362.32 ± 18.43
